# The study of social participation and its influences on young and middle-aged patients with myasthenia gravis

**DOI:** 10.3389/fneur.2025.1603145

**Published:** 2025-10-14

**Authors:** Juan Chen, Lengmeng Wang, Yan Fang, Yanhong Pan, Lin Lin, Lifeng Zhang

**Affiliations:** ^1^The First Affiliated Hospital of Fujian Medical University, Fuzhou, Fujian, China; ^2^National Regional Medical Center, Fudan University Affiliated Huashan Hospital Fujian Hospital, The First Affiliated Hospital of Fujian Medical University (Binhai Campus), Fuzhou, Fujian, China; ^3^Department of Paediatrics, Shenzhen Longhua Maternity and Child Healthcare Hospital, Shenzhen, Guangdong, China; ^4^Department of Nursing, The First Affiliated Hospital of Fujian Medical University, Fuzhou, Fujian, China; ^5^School of Nursing, Sun Yat-sen University, Guangzhou, Guangdong Province, China

**Keywords:** myasthenia gravis, young and middle-aged, social participation, depression, family functioning, social support

## Abstract

**Objective:**

To describe the social participation of young and middle-aged patients with myasthenia gravis and explore the main influences to provide a basis for improving social participation behavior.

**Methods:**

From January 2020 to December 2022, 145 patients with myasthenia gravis aged between 18 and 60 years who were recruited from a Grade A tertiary hospital in Shenzhen were selected via a convenience sampling method. A total of 145 patients with nonmyasthenia gravis aged between 18 and 60 years who received physical examination at the same hospital at the same time as the control group were included. A general information questionnaire, Assessment of Life Habits, the Beck Depression Inventory, the Family Assessment Device, and the Perceived Social Support Scale were used to evaluate the survey. The results The total social participation score of young and middle-aged patients with myasthenia gravis was 88.90 ± 33.05. Among them, three dimensions with higher scores were independent participation in social life and social relations, activity, and self-care. Compared with those of the same age group, the social participation scores of young and middle-aged patients with myasthenia gravis were higher than those of the control group, who were in activities, independently participated and helped and supported others, indicating that their level of social participation was lower than that of the control group. Multiple linear regression revealed that the level of social participation in female patients who were unemployed, lived in rural areas, needed daily care, had a long disease course, had weak daily activity ability, had depression, and had poor family functioning was relatively low (*p* < 0.001, *R^2^ =* 0.527).

**Conclusion:**

The level of social participation of young and middle-aged myasthenia gravis patients was relatively low and far lower than that of the control group, especially in terms of independent participation in social life and social interactions, activities and self-care, which needs to be improved.

## 1 Introduction

Myasthenia gravis (MG) is an acquired autoimmune disorder of neuromuscular transmission caused by autoantibodies (1). MG can be classified into two major clinical forms: ocular MG (OMG), in which symptoms are limited to the extraocular and levator palpebrae muscles (such as ptosis and diplopia); and generalized MG (gMG), in which muscle weakness extends to the face, limbs, pharyngeal, and respiratory muscles with varying severity. OMG often presents as the initial manifestation and may progress to the generalized form in a proportion of patients, whereas gMG involves a broader range of muscle groups and can be life-threatening (2). Its progression may lead to respiratory dysfunction, asphyxiation, and other severe complications that pose a significant threat to patients’ health (3, 4). In China, the annual incidence of MG is 0.68 per 100,000 individuals (5). MG can lead to long-term limitations in physical activity and fatigue. By contrast, global incidence estimates range from approximately 0.17 to 3.0 per 100,000 person-years, with some regions reporting 0.3 to 2.8 cases per 100,000 (6), while in the United States the incidence is about 3.2 per 100,000 and the prevalence reaches approximately 37 per 100,000 (7). Globally, the overall prevalence of MG is estimated at around 20 per 100,000 population, and the disease disproportionately affects young adult women and older men—adding emphasis to the significant disease burden in young and middle-aged adults, which strengthens the rationale for focusing on social participation and quality-of-life impacts in this demographic (8). Additionally, there is a high prevalence of negative emotions such as depression and stigma among patients (9). Moreover, the treatment costs and impact on employment can further increase the burden on families (10).

With shifts in disease profiles and changes in lifestyle, the onset age of MG is trending younger (11). Many young and middle-aged patients are diagnosed during a critical stage of their careers. As the number of affected individuals in this age group increases, the impact of MG on social participation has garnered increasing attention (12). This demographic not only represents the primary labor force but also serves as the economic and emotional backbone of families. When faced with loss of labor capacity, reduced income, and increased medical expenses, both the physical and mental well-being of these patients are profoundly affected (13). Their social participation is also significantly impacted. In the International Classification of Functioning framework, social participation denotes involvement in real-life situations (e.g., employment, household, interpersonal, and civic domains). Recent NMD-focused evidence suggests that participation-oriented rehabilitation is a key, patient-valued target and may improve mobility, self-care and social participation, even though the certainty of evidence remains limited (14). Patient-reported international datasets further show that people with MG explicitly prioritize returning to work/school, resuming physical and social activities, and reducing the day-to-day limitations that impede participation (10, 15).

Emerging multinational studies now quantify how MG severity translates into activity and participation restrictions. In a real-world physician-reported cohort from the US and five European countries, higher MGFA class correlated with greater impairment in activities of daily living (ADL) (16). Complementary multinational analyses using the Work Productivity and Activity Impairment instrument show that MG severity is associated with substantial productivity losses (absenteeism, presenteeism and early retirement) and with impairment of non-work activities (17, 18). Together, these data indicate that participation—especially employment—declines as symptoms worsen, underscoring the need to assess and support social participation among working-age adults with MG (16–18).

Situating this question in China adds public-health and health-policy relevance. Nationwide analyses have characterized MG’s incidence, in-hospital mortality and economic burden in mainland China, and recent registry-based work shows substantial out-of-pocket and societal costs, with disease severity driving both direct and indirect costs (19, 20). Chinese cohorts also report marked decrements in quality of life among adults with MG, consistent with participation limitations reported internationally (21). However, few studies have specifically examined social participation (e.g., labor-market engagement and community involvement) among young and middle-aged Chinese adults with MG. Addressing this gap can enrich the international literature and highlight the originality and policy significance of focusing on China’s working-age population (19–21).

This study aims to describe the current state of social participation among young and middle-aged patients with MG and explore its influencing factors, in order to provide a theoretical basis for designing rehabilitation interventions that promote social reintegration and improve quality of life. The findings are expected to inform the development of targeted health policies, rehabilitation programs, and psychosocial support services for this patient population.

## 2 Subjects and methods

### 2.1 Study subjects

Using convenience sampling, we selected MG patients who attended follow-up visits at the neurology and thoracic surgery outpatient departments of a tertiary hospital in Shenzhen between January 2020 and December 2022 (hereafter referred to as the “MG group”). A control group (“non-MG group”) was established by recruiting individuals without MG who underwent physical examinations at the same hospital during the same period. The inclusion and exclusion criteria were designed to ensure comparability between groups while minimizing confounding factors.

Inclusion criteria for the MG group:

① Patients meeting the clinical classification criteria of the American Myasthenia Gravis Association and the Myasthenia Gravis Quantitative Score (MGS), as well as CT and MRI examination standards, with a confirmed clinical diagnosis of MG and an MGS ranging from 0 to 26 (4). The MGS range of 0–26 was selected to include patients with mild to moderate disease severity, ensuring that participants retained sufficient cognitive and communicative abilities to complete the questionnaires reliably. This range excludes only the most severe cases (MGS > 26), which are often associated with critical impairment and may not be representative of the broader MG population in social participation studies.
② Age ≥18 years and <60 years.
③ A confirmed diagnosis for at least 3 months to ensure disease stability and adaptation.
④ A minimum level of primary school education, with adequate reading and writing skills, clear consciousness, and normal communication ability, enabling patients to complete the questionnaire independently or with minimal assistance.

Inclusion criteria for the non-MG group:

① Age ≥18 years and <60 years.
② A minimum of primary school education.
③ Provision of informed consent and voluntary participation in the study.
④ Absence of any chronic physical or mental conditions that could significantly impair social participation (e.g., diabetes, hypertension, chronic pain syndromes, neurological disorders other than MG, or severe psychiatric conditions), as confirmed through self-report and medical record review.

Exclusion criteria for both groups:

① Patients with malignant tumors or those in the terminal stages of other diseases.
② Patients with mental illnesses or those currently taking related medications.
③ Patients with severe communication barriers, making it difficult to accurately understand or respond to the questionnaire.
④ For the non-MG group, individuals with any chronic condition that could substantially affect social participation were also excluded to reduce potential confounding.

To enhance comparability, the non-MG group was matched 1:1 with the MG group based on age and sex. While other sociodemographic factors (e.g., education, marital status, employment) were not explicitly matched during recruitment, these variables were recorded and later controlled for in the statistical analysis to ensure that any observed differences in social participation were attributable to MG status rather than demographic disparities.

According to the requirements for multiple linear regression analysis, the sample size should be 5–10 times the number of independent variables. In this study, with 14 variables and considering a 5% invalid questionnaire rate, the required sample size was 74–147 cases. A total of 151 questionnaires were distributed to the MG group, with 145 valid responses (96.0% response rate), and 155 questionnaires were distributed to the non-MG group, with 145 valid responses (93.5% response rate). All participants voluntarily participated and signed informed consent forms. The study was approved by the hospital’s ethics committee [Approval No. Lun (2022) 145].

### 2.2 Measurement instruments

#### 2.2.1 General demographic questionnaire

A questionnaire was designed by the researchers to collect the following information:

Sociodemographic data: Age, sex, educational level, marital status, occupation, employment status, per capita monthly household income (in yuan), method of medical expense payment, monthly medical expenditure (in yuan), residence, living conditions, frequency of housework, and daily activity ability.

Items such as “frequency of housework” and “living conditions” were included based on their established relevance to social participation in previous research. Frequency of housework reflects the patient’s ability to perform instrumental activities of daily living (IADLs), which is a key component of social participation (22). Living conditions (e.g., living alone vs. with others) may influence social support networks and opportunities for social engagement (23).

Disease-related information: Clinical classification, disease duration, primary symptoms, MG quantitative score, main treatment measures, complications, and frequency of hospitalizations.

#### 2.2.2 Social participation scale

Originally developed by Fougeyrollas et al. (12) and later translated into Chinese by Zeng et al. (13), this scale comprises eight dimensions: physical activity (4 items), self-care (5 items), activities (6 items), financial management (1 item), leisure (1 item), independent participation in social life and social relationships (6 items), helping and supporting others (1 item), and the ability to live according to one’s desired lifestyle (1 item), for a total of 25 items. The scale uses a 5-point Likert scoring system (0–4 points per item) with a total score range of 0–100, where lower scores indicate a higher level of social participation. Based on the original validation study and subsequent clinical applications, total scores are typically interpreted as follows: 0–33 indicates high social participation, 34–66 indicates moderate social participation, and 67–100 indicates low social participation (8, 9). The Chinese version of the scale has demonstrated good construct validity in previous studies involving patients with chronic conditions, with confirmatory factor analysis supporting the original eight-factor structure (*χ*^2^/df = 2.456, RMSEA = 0.066, CFI = 0.912, GFI = 0.904) (24). Criterion validity was established through significant correlations with measures of physical function (*r* = 0.52) and mental health (*r* = −0.48) in a sample of Chinese stroke patients (25). In this study, the Cronbach’s *α* coefficient for the scale was 0.896.

#### 2.2.3 Beck depression inventory

Developed by Mostafa Alim et al. (26) and translated into Chinese by Yang et al. (27), this inventory consists of 21 items, each scored on a 4-point Likert scale (0–3 points), yielding a total score of 0–63. Higher scores denote more severe depressive symptoms. The score interpretation is as follows: 0–13 indicates no depression; 14–19 indicates mild depression; 20–28 indicates moderate depression; and scores above 29 indicate severe depression. The Chinese version has demonstrated strong psychometric properties in various populations. In a study involving Chinese, the scale showed excellent internal consistency (Cronbach’s *α* = 0.925) and good item-total correlations (*r* = 0.492–0.761) (28). Exploratory and confirmatory factor analyses supported a three-factor structure (emotional, somatic, and negative symptoms) with satisfactory model fit (*χ*^2^/df = 1.604, CFI = 0.940, TLI = 0.932, RMSEA = 0.051) (28). Criterion validity was established against clinical diagnoses, with ROC analysis showing an AUC of 0.904 for distinguishing severe depression, and a cutoff score of 17 providing sensitivity of 0.86 and specificity of 0.88 in Chinese patient populations (29). In this study, the Cronbach’s *α* coefficient was 0.893.

#### 2.2.4 Family function evaluation scale

Developed by Epstem et al. (30) and translated and revised by Liu et al. (31), this scale comprises seven dimensions: problem-solving ability (6 items), communication efficiency (9 items), role identification (11 items), emotional response ability (6 items), emotional involvement (7 items), behavioral control (9 items), and overall family functioning (12 items), totaling 60 items. It employs a 4-point Likert scale with responses ranging from “very similar” to “not at all similar” (scored 1–4), resulting in a total score range of 60–240; lower scores indicate better family functioning. The Chinese version has been validated in multiple studies. Internal consistency for the total scale is good (Cronbach’s *α* = 0.808), and subscale α values range from 0.603 to 0.729 (32). Test–retest reliability over a 21-day interval was 0.74 (*p* < 0.01) (17). Construct validity was supported by confirmatory factor analysis, which confirmed a three-factor model with acceptable fit indices (32). Criterion validity was established through significant correlations with the General Functioning subscale of the McMaster Family Assessment Device (*r* = 0.72, *p* < 0.001) (33). The Cronbach’s *α* coefficient in this study was 0.808.

#### 2.2.5 Perceived social support scale

Originally developed by Zimet et al. (34) and later simplified and revised by Jiang Qianjin et al. (35), this scale consists of 12 items across three dimensions: family support (4 items), friend support (4 items), and other support (4 items). It uses a 7-point Likert scale ranging from “strongly disagree” (1 point) to “strongly agree” (7 points), with a total score ranging from 12 to 84; higher scores indicate greater levels of perceived social support. The Chinese version has shown robust psychometric properties in medical populations. The three-factor structure was confirmed through exploratory and confirmatory factor analyses, with a cumulative variance explanation of 67.139% and satisfactory model fit (*χ*^2^/df = 3.534, GFI = 0.866, RMSEA = 0.113) (36). Internal consistency is high, with Cronbach’s *α* values of 0.840 for the total scale and 0.813–0.820 for subscales (36). The scale also demonstrates good content validity, with subscale-total correlations ranging from 0.713 to 0.770 (36). In this study, the Cronbach’s α coefficient was 0.902.

### 2.3 Data collection methods

The researcher conducted face-to-face questionnaire surveys via standardized measurement tools and uniform instructions. To minimize social desirability bias, all participants were assured of anonymity and confidentiality, and researchers provided neutral, non-leading instructions throughout the process. Researchers ensured that each patient fully understood all sections of the questionnaire before completing it on the spot. When assistance was required, it was limited to reading items aloud or explaining question meanings without suggesting or influencing responses, thus maintaining response validity. Upon completion, the questionnaires were immediately collected and reviewed by the researcher for completeness. Any missing, omitted, or erroneous responses were promptly supplemented or corrected.

### 2.4 Statistical analysis methods

**Figure 1:**
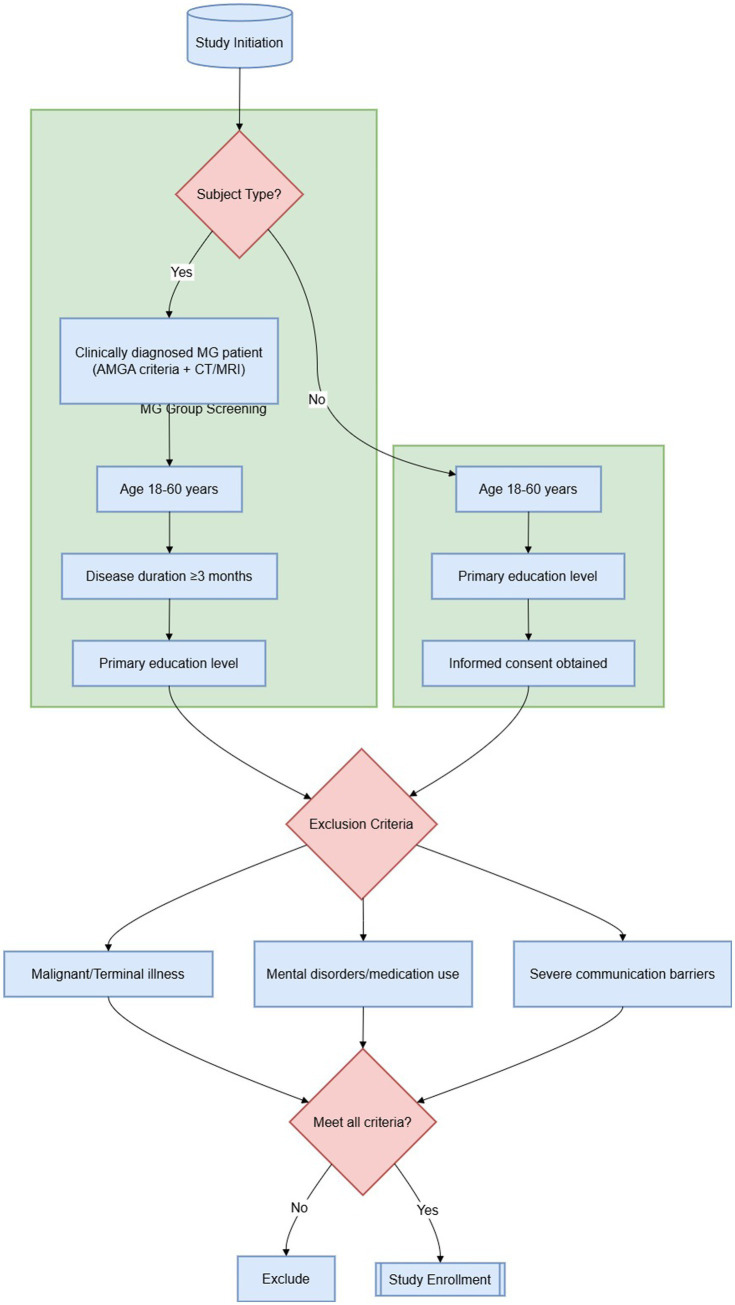
Inclusion and exclusion flowchart.

The data were analyzed via SPSS version 25.0. Continuous variables are presented as the means ± standard deviations, whereas categorical variables are described as frequencies and percentages. The normality of continuous variables was assessed using the Shapiro–Wilk test, and homoscedasticity was verified using Levene’s test before conducting parametric tests. Group comparisons were performed via t tests and chi-square tests. Univariate analyses of social participation in the MG group were conducted via t tests and one-way ANOVA across different variables. For multiple comparisons, Bonferroni correction was applied to control the family-wise error rate. Pearson correlation analysis was used to assess the relationships between social participation and variables such as the MG quantitative score, modified Barthel Index score, depression, family functioning, and social support. Variables with *p* < 0.05 in the univariate analysis were subsequently included in a multiple linear regression analysis (using the Enter method) to identify factors influencing social participation among MG patients. Collinearity among independent variables was assessed using variance inflation factor (VIF) and tolerance statistics, with VIF < 5 and tolerance > 0.2 indicating no substantial multicollinearity. The Enter method was chosen over stepwise methods due to its theoretical grounding and to avoid the well-documented limitations of data-driven variable selection. Model fit was assessed using the coefficient of determination (*R*^2^) and adjusted R^2^. Although questionnaires were reviewed at the time of collection and missing data were minimal (<2%), any missing values were handled using listwise deletion to ensure complete case analysis (Figure 1).

## 3 Results

### 3.1 Comparison of the general characteristics of the study subjects

In the MG group, 151 questionnaires were distributed, and 145 valid questionnaires were collected, yielding an effective response rate of 96.0%. In the non-MG group, 155 questionnaires were distributed, and 145 valid questionnaires were collected, yielding an effective response rate of 93.5%. A comparison of the general characteristics between the two groups revealed no statistically significant differences in age or sex composition (*p* > 0.05). However, significant differences were observed between the groups in terms of educational level, employment status, occupation, residence, living conditions, per capita monthly household income (Yuan), method of payment for medical expenses, monthly medical expenditure (Yuan), understanding of one’s own disease, primary reasons for limitations in undertaking household responsibilities, engagement in outdoor exercise, frequency of outdoor activities, total duration of outdoor activities (hours per week), and whether assistance was required for outdoor activities (*p* < 0.05; Table 1).

**Table 1:** Comparison of the general characteristics of the study participants (*n* = 290).

Variable	MG group (*n*₁ = 145)	Non-MG group (*n*₂ = 145)	*t*/*χ*^2^	*p*
	*n* (%)/Mean ± SD	*n* (%)/Mean ± SD		
Age (years)	37.26 ± 11.13	37.3 ± 11.09	-0.081ᵃ	0.935
18–29	33 (22.7%)	33 (22.7%)		
30–44	83 (59.3%)	83 (59.3%)		
45–60	29 (20.0%)	29 (20.0%)		
Gender			0.007ᵇ	0.932
Male	47 (32.4%)	47 (32.4%)		
Female	98 (67.6%)	98 (67.6%)		
Education level			27.748ᵇ	<0.001***
High school or below	52 (35.9%)	26 (18.0%)		
College	54 (37.2%)	36 (24.8%)		
Bachelor’s or above	39 (26.9%)	83 (57.2%)		
Employment status			30.650ᵇ	<0.001***
Nonemployed	54 (37.2%)	15 (10.4%)		
Full-time	71 (49.0%)	105 (72.4%)		
Part-time	20 (13.8%)	25 (17.2%)		
Residential environment			30.377ᵇ	<0.001***
Rural	27 (18.6%)	24 (16.5%)		
Urban	118 (81.4%)	121 (83.5%)		
Living arrangement			18.924ᵇ	<0.001***
Living alone	14 (9.7%)	34 (23.4%)		
With family/friends	131 (90.3%)	111 (76.6%)		
Monthly household income (CNY)			19.632ᵇ	<0.001***
<5,000	66 (45.5%)	45 (31.0%)		
5,000–10,000	60 (41.4%)	48 (33.1%)		
>10,000	19 (13.1%)	52 (35.9%)		
Monthly medical expenses (CNY)			40.531ᵇ	<0.001***
<1,000	49 (33.8%)	97 (66.8%)		
1,001–8,000	96 (66.2%)	48 (33.2%)		
Disease awareness			18.453ᵇ	<0.001***
Aware	66 (45.5%)	110 (75.9%)		
Unaware	79 (54.5%)	35 (24.1%)		
Primary reason for household burden			41.611ᵇ	<0.001***
Physical health issues	75 (51.7%)	23 (15.9%)		
No financial burden	37 (25.5%)	25 (17.2%)		
Lack of interest/necessity	24 (16.6%)	30 (20.7%)		
Negative emotions	9 (6.2%)	67 (46.2%)		
Engaged in outdoor exercise			8.450ᵇ	0.004**
No	37 (25.5%)	60 (41.4%)		
Yes	108 (74.5%)	85 (58.6%)		
Outdoor activity frequency			16.030ᵇ	0.007**
≥Daily	10 (6.9%)	16 (11.0%)		
Every 2–3 days	50 (34.5%)	37 (25.5%)		
Every 4–5 days	42 (29.0%)	51 (35.2%)		
Every 5–7 days	26 (17.9%)	17 (11.7%)		
>7 days	17 (11.7%)	24 (16.6%)		
Total outdoor activity duration (hours/week)			41.500ᵇ	<0.001***
<7	36 (24.8%)	88 (60.7%)		
7–56	109 (75.2%)	57 (39.3%)		
Require assistance for outdoor activities			31.568ᵇ	<0.001***
No	65 (44.8%)	112 (77.2%)		
Yes	80 (55.2%)	33 (22.8%)		

ᵃ*t*-test; Chi-square test. *p < 0.05; **p < 0.01; ***p < 0.001.

### 3.2 Disease characteristics of young and middle-aged MG patients

This study included a total of 145 young and middle-aged MG patients. Among them, 65.5% were classified as having generalized myasthenia gravis, and 55.2% had a disease course exceeding 5 years. The predominant initial symptoms at diagnosis were ptosis (35.2%) and generalized weakness (42.8%). Similarly, the current primary symptoms remained ptosis (34.5%) and generalized weakness (33.8%). The mean Myasthenia Gravis Quantitative Score (MGQS) was 8.26 ± 7.78. Pharmacological treatment was administered to 96.6% of patients, with 95.7% receiving acetylcholinesterase inhibitors (e.g., pyridostigmine). Regarding follow-up, 73.1% attended regular visits, with 50.3% having appointments every 3 months. The main reason for irregular follow-up was forgetfulness (46.2%). Nearly half of the patients (49.0%) reported experiencing pain, primarily located in the trunk (62.0%). Comorbidities were present in 27.6% of patients, with thyroid disorders being the most common (87.5%). Additionally, 29.7% had been hospitalized due to MG, and 17.2% had experienced a myasthenic crisis. Details are provided in Table 2.

**Table 2:** Disease characteristics of young and middle-aged mg patients (*n* = 145).

Variable	*n* (%)/ Mean ± SD	Variable	*n* (%)
MGFA clinical type		Follow-up Frequency	
Ocular MG	50 (34.5)	Monthly	18 (12.5)
Generalized MG	95 (65.5)	Every 3 months	73 (50.3)
Disease duration (years)		Every 6 months	37 (25.5)
<5	65 (44.8)	Yearly or less	17 (11.7)
5–30	80 (55.2)	Reason for No Regular Follow-up	
Initial major symptom		Too busy	39 (26.9)
Ptosis	51 (35.2)	Forgot	67 (46.2)
Limb weakness	29 (20.0)	Negative psychology	31 (21.4)
Generalized weakness	62 (42.8)	Objective reasons	8 (5.5)
Dyspnea	3 (2.0)	Experience of Pain	
Current major symptom		No	74 (51.0)
Ptosis	50 (34.5)	Yes	71 (49.0)
Limb weakness	37 (25.5)	Pain Location (n = 71)	
Generalized weakness	49 (33.8)	Head	10 (14.1)
Dyspnea	9 (6.2)	Trunk	44 (62.0)
MG quantitative score	8.26 ± 7.78	Limbs	17 (23.9)
Primary treatment		Comorbidities	
Surgery only	5 (3.4)	No	105 (72.4)
Medication + Surgery	16 (11.1)	Yes	40 (27.6)
Medication only	124 (85.5)	Type of Comorbidity (n = 40)	
Medication type (Multiple choices, *n* = 140)		Hyperthyroidism	24 (60.0)
Immunosuppressants	54 (38.6)	Hypothyroidism	11 (27.5)
AChE inhibitors (e.g., Pyridostigmine)	134 (95.7)	Thymoma	5 (12.5)
Corticosteroids	51 (36.4)	Hospitalization due to MG	
Regular follow-up		No	102 (70.3)
No	39 (26.9)	Yes	43 (29.7)
Yes	106 (73.1)	Myasthenic Crisis	
		No	120 (82.8)
		Yes	25 (17.2)

### 3.3 Comparison of social participation between study groups

The results indicated that the MG group had significantly higher scores in overall social participation across all dimensions than did the non-MG group (*p* < 0.05) (Table 3).

**Table 3:** Comparison of social participation (*n* = 290).

Variable	MG group (*n*₁ = 145)	Non-MG group (*n*₂ = 145)	*t*	*p*
	Mean ± SD	Mean ± SD		
Total social participation score	88.90 ± 33.05	48.17 ± 54.07	6.680	<0.001***
Subscale scores				
Physical mobility	5.14 ± 3.10	3.10 ± 3.93	4.315	<0.001***
Self-care	8.73 ± 5.05	5.24 ± 6.64	4.038	<0.001***
Activities	12.88 ± 4.45	6.48 ± 7.78	7.289	<0.001***
Financial management	2.20 ± 0.97	1.08 ± 1.39	7.032	<0.001***
Leisure	1.99 ± 1.10	1.07 ± 1.37	5.417	<0.001***
Social life and relationships	13.16 ± 4.61	6.92 ± 7.23	7.800	<0.001***
Helping/supporting others	2.50 ± 0.95	1.15 ± 1.30	9.098	<0.001***
Living as desired	2.41 ± 1.06	1.39 ± 1.38	6.412	<0.001***

***p < 0.001.

### 3.4 Univariate analysis of social participation in the MG

Univariate analysis revealed statistically significant differences in social participation scores based on sex, occupation, employment status, monthly medical expenditure (in yuan), place of residence, whether daily care is needed, total duration of outdoor activities (hours per week), level of understanding of one’s own disease, disease duration (in years), and the presence of pain (*p* < 0.05; Table 4).

**Table 4:** Univariate analysis of social participation in the MG group (*n*₁ = 145).

Variable	Social participation score			
	*n*	Mean ± SD	*t*/*F*	*p*
Gender			−2.271ᵗ	0.025*
Male	47	44.21 ± 15.32		
Female	98	51.33 ± 18.67		
Occupation			2.703ᶠ	0.012*
Civil servant/clerical	102	45.08 ± 18.53		
Professional (teacher, doctor)	17	48.15 ± 13.42		
Farmer/worker	10	49.96 ± 21.38		
Other occupationsᵃ	16	35.4 ± 19.25		
Employment Status			2.296ᶠ	0.023*
Nonemployed	54	53.39 ± 17.36		
Full-time	71	46.43 ± 18.13		
Part-time	20	47.23 ± 16.87		
Monthly Medical Expenses (CNY)			−2.558ᵗ	0.012*
<1,000	49	43.80 ± 21.54		
1,001–8,000	96	51.69 ± 15.18		
Residence			3.192ᵗ	0.026*
Rural	27	40.70 ± 23.73		
Urban	118	49.76 ± 16.06		
Require Daily Care			−2.604ᵗ	0.010*
No	111	46.92 ± 18.15		
Yes	34	55.88 ± 15.44		
Outdoor activity duration (hours/week)			−2.318ᵗ	0.022*
<7	36	43.11 ± 21.86		
7–56	109	50.97 ± 16.04		
Disease awareness			3.387ᵗ	0.037*
Aware	66	41.39 ± 20.12		
Unaware	79	53.78 ± 25.85		
Disease duration (years)			−2.010ᵗ	0.046*
<5	65	45.74 ± 17.30		
5–30	80	51.69 ± 18.06		
Experience pain			−2.273ᵗ	0.025*
No	74	45.76 ± 19.31		
Yes	71	52.42 ± 15.75		

ᵃOther occupations: divorced, widowed; *p < 0.05.

### 3.5 Correlation analysis between social participation and the MG quantitative score, modified Barthel index, depression, family function, and social support in the MG group

Correlation analysis revealed that the MG quantitative score, depression score, and family function score were significantly positively correlated with the social participation score (*p* < 0.05). In contrast, the modified Barthel index score was significantly negatively correlated with the social participation score (*p* < 0.001; Table 5).

**Table 5:** Correlation analysis of social participation in the MG group.

Variable	Social participation score	
	Mean ± SD (points)	*r*
MG quantitative score	8.26 ± 7.78	0.175*
Modified Barthel index score	70.04 ± 25.41	−0.495***
Depression score	34.96 ± 11.22	0.531**
Family function score	157.80 ± 3.26	0.248**
Social support score	59.77 ± 10.18	0.154

### 3.6 Multivariate analysis of factors influencing social participation in the MG group

Variables that were statistically significant in both the univariate and correlation analyses were entered as independent variables, with the social participation score as the dependent variable, via the Enter method. The criteria for entry and removal were set at 0.05 and 0.10, respectively, with variable assignments, as shown in Table 6. The results revealed that sex, place of residence, daily care, disease duration, modified Barthel index, depression, and family function were significant influencing factors for social participation (*p* < 0.001; Table 7).

**Table 6:** Variable assignment.

Variable	Assignment
Gender	Male = 1, Female = 2
Occupation	
(Reference: Civil servant/clerical)	Civil servant/clerical (A1 = 0, A2 = 0, A3 = 0)
Professional (teacher, doctor)	A1 = 1, A2 = 0, A3 = 0
Farmer/worker	A1 = 0, A2 = 1, A3 = 0
Other occupations	A1 = 0, A2 = 0, A3 = 1
Employment status	
(Reference: Nonemployed)	Nonemployed (B1 = 0, B2 = 0)
Full-time	B1 = 1, B2 = 0
Part-time	B1 = 0, B2 = 1
Monthly medical expenses (CNY)	<1,000 = 1, 1,001–8,000 = 2
Residence	Rural = 1, Urban = 2
Require daily care	No = 1, Yes = 2
Outdoor activity duration (hours/week)	<7 = 1, 7–56 = 2
Disease awareness	Aware = 1, Unaware = 2
MBI total score	Raw value entered
Disease duration (years)	<5 = 1, 5–30 = 2
MG quantitative score	Raw value entered
Experience pain	No = 1, Yes = 2
Depression score	Raw value entered
Family function score	Raw value entered

**Table 7:** Multiple linear regression analysis of social participation in the MG (*n*₁ = 145).

Model	Unstandardized coefficients		Standardized coefficients	*t*	*p*
	B	SE	*β*		
(Constant)	1.501	0.306	–	4.911	<0.001
Gender	0.165	0.078	0.157	2.132	0.035*
Occupation	0.825	0.698	0.092	1.183	0.239
Employment status	1.530	1.519	0.079	1.007	0.316
Monthly medical expenses	−0.563	1.396	−0.030	−0.404	0.687
Residence (Ref: Rural)					
Urban	3.955	1.310	0.208	3.019	0.003**
Require daily care (Ref: No)					
Yes	8.963	3.442	0.213	2.604	0.010*
Outdoor activity duration	−1.941	1.983	−0.071	−0.979	0.330
Disease awareness	−2.260	1.164	−0.133	−1.940	0.055
Disease duration (Ref: <5 years)					
5–30 years	2.472	2.360	0.069	0.873	0.014*
MG quantitative score	0.108	0.163	0.047	0.663	0.509
Modified Barthel index	−6.662	1.438	−0.325	−4.634	<0.001**
Depression	13.131	1.751	0.531	7.501	<0.001**
Family function	8.954	2.928	0.248	3.057	0.003**

Dependent variable: Social participation score. *R* = 0.725, *R*^2^ = 0.527, Adjusted *R*^2^ = 0.468, *F* = 8.847, *p* < 0.001. *p < 0.05; **p < 0.01.

## 4 Discussion

### 4.1 Summary of main findings

This study provides comprehensive insights into the social participation of young and middle-aged patients with MG and its influencing factors. Our results demonstrate that this population experiences significantly impaired social participation, with a total score of 88.90 ± 33.05 indicating low engagement levels. Based on the standardized regression coefficients (*β* values) from our multiple linear regression analysis, depressive symptoms (*β* = 0.531, *p* < 0.001) emerged as the strongest predictor of impaired social participation, followed by daily activity ability as measured by the modified Barthel Index (*β* = −0.325, p < 0.001). Family functioning (*β* = 0.248, *p* = 0.003) and urban residence (*β* = 0.208, p = 0.003) also demonstrated substantial effects, while the need for daily care showed a moderate effect (*β* = 0.213, *p* = 0.010). Gender (*β* = 0.157, *p* = 0.035) and disease duration (*β* = 0.069, *p* = 0.014) had smaller but statistically significant effects (37). The strong impact of depression underscores its central role in limiting social engagement, potentially due to its effects on motivation, energy levels, and self-perception. The significant contribution of physical functioning highlights the importance of addressing mobility and self-care limitations to facilitate social participation. These findings suggest that interventions targeting both psychological well-being and physical functioning may have the greatest potential to improve social participation outcomes in this population.

### 4.2 Comparison with previous studies

The observed low level of social participation among young and middle-aged MG patients is consistent with previous domestic studies (10, 11). International cohorts likewise show marked restrictions in role and social participation among MG patients; for example, in the multinational MyRealWorld-MG study, one-third of respondents reported a work or study impact within the past month, and difficulties with work/housework were commonly rated as severe (15). The prolonged course of MG, characterized by persistent symptoms and repeated treatments that may progressively deteriorate patients’ conditions, appears to erode self-confidence and limit social engagement. Findings from a large German case–control study also document reduced physical and mental health domains and highlight lower activities of daily life and limited social well-being as key contributors to poorer quality of life (38). Specifically, patients exhibited particular difficulties in the dimensions of independent involvement in social life and social relationships, self-care, and general activities, mirroring patterns observed in other chronic neurological conditions. Our study also found that social participation in this population is significantly affected by depressive symptoms (*p* < 0.01), with the influence being somewhat greater than that reported in studies on poststroke patients (39). Consistent with this, international data indicate that roughly 31–43% of MG patients screen positive for depressive symptoms, and depression independently predicts worse health-related quality of life and participation limitations (15, 40).

### 4.3 Psychosocial factors influencing social participation

#### 4.3.1 Demographic and clinical factors

The study revealed that young and middle-aged female MG patients presented lower levels of social participation. This may be attributed to generally lower psychological resilience among women, making them more susceptible to negative emotions and more likely to avoid outdoor activities (41). Moreover, disease-related sequelae that alter appearance may deter female patients, who often place high value on their physical image, from engaging in social activities (42).

Young and middle-aged MG patients residing in rural areas demonstrated lower social participation. Factors such as lower employment rates, job instability, poor job quality, and an underdeveloped social security system in rural regions, compounded by the physical limitations of MG patients, preclude them from engaging in heavy manual labor, resulting in fewer employment opportunities (43).

Patients with diminished daily activity abilities had lower social participation scores. Owing to their limited mobility and poor self-care capacity, these patients often depend heavily on caregivers for basic needs. Their restricted ability to engage in social activities, coupled with weakened family and social roles, can lead to feelings of shame and self-loathing (44, 45).

The duration of the disease also impacts social participation. Patients with a prolonged and recurrent course may lose confidence in their ability to participate socially, leading to negative emotions that further diminish their engagement (46).

#### 4.3.2 Psychological and family factors

Depression in MG patients may stem not only from the adverse effects of long-term immunotherapy but also from the fear and stress associated with the disease itself, leading to social withdrawal and isolation.

Family functioning significantly influences social participation (*p* < 0.01). Better family functioning, greater family support, closer interpersonal relationships, and greater mutual understanding and support among family members can enhance patients’ social participation (47).

### 4.4 Clinical implications and recommendations

Our findings underscore the need for comprehensive approaches to improve social participation in MG patients. In the short term, if patients do not experience significant symptom relief after intervention, they may develop depressive feelings and doubt the effectiveness of caregiving. The lack of affirmation and support from family and friends further hinders their social participation.

Clinical staff should encourage patients to actively adhere to both inpatient treatments and postdischarge medication regimens to control the disease. Efforts should be made to delay disease progression through optimal management. We recommend implementing evidence-based interventions including community-based rehabilitation programs specifically tailored for MG patients. Cognitive-behavioral therapy adapted for chronic illness management should be incorporated into treatment plans. Telemedicine or digital follow-up systems can improve treatment adherence and social participation, particularly for patients in remote areas. Family-centered interventions are essential to enhance support systems and improve overall outcomes. Regular physical function assessment and early rehabilitation interventions should be implemented to maintain patients’ functional abilities.

Healthcare providers should pay particular attention to the psychological care of female patients and those from rural areas, as these groups demonstrate particular vulnerabilities. For patients with diminished daily activity abilities, prompt assessment and implementation of rehabilitation interventions are essential. Regular monitoring through follow-up appointments helps patients maintain their optimal functional status and supports their engagement in social activities.

### 4.5 Study limitations and strengths

This study has several limitations that should be considered. First, the use of convenience sampling from a single tertiary hospital may limit the generalizability of our findings and introduces potential selection bias, as participants may not be fully representative of the broader MG population. While random sampling would have been methodologically preferable, it was not feasible due to the relatively low prevalence of MG and the challenges in accessing a complete patient registry. Second, the cross-sectional design prevents us from establishing causal relationships between the identified factors and social participation. Third, self-report measures may be subject to recall and social desirability biases. Finally, our findings may be influenced by unmeasured confounding factors such as comorbidities, healthcare access disparities, and cultural factors that could affect social participation but were not assessed in this study. Despite these limitations, our study has several notable strengths, including the use of a matched control group, scales used in this study have been rigorously translated and validated in Chinese populations, demonstrating strong psychometric properties in patients with chronic health conditions. The Chinese versions of these scales have shown appropriate factor structures, high internal consistency, and significant correlations with relevant health outcomes (10, 16–19). Although no validation study has been conducted specifically in Chinese MG patients, the high reliability observed in our sample (Cronbach’s *α* values between 0.808 and 0.902) and the previous validation work in similar chronic illness populations support their cultural and clinical applicability for this study. Future research should directly examine the psychometric properties of these scales in Chinese MG populations, and focus on the understudied population of young and middle-aged MG patients, which provides valuable insights for developing targeted interventions.

### 4.6 Future research directions

Future research should address these limitations through longitudinal designs to track changes in social participation over the disease course and establish causal pathways. Intervention trials examining the effectiveness of structured rehabilitation programs, psychoeducational interventions, and family support programs are needed to develop evidence-based practices. Additionally, future studies should include more diverse samples across multiple centers using stratified random sampling methods to enhance representativeness and incorporate cultural and occupational variables to better understand the urban/rural disparities in social participation outcomes. Multi-center collaborative studies would be particularly valuable for obtaining larger, more representative samples and improving the generalizability of findings.

## 5 Conclusion

Social participation levels among young and middle-aged MG patients are suboptimal. Clinically, attention should be directed toward patients’ engagement in self-care, general activities, and independent social life and relationships. Special focus should be given to female patients, those from rural areas, patients who require daily care, and those with weak daily activity abilities, longer disease durations, more severe depressive symptoms, and poorer family functioning. Health education, psychological support, and active family involvement may improve social participation levels in this patient population. Importantly, this study investigated only young and middle-aged MG patients attending a single tertiary hospital outpatient department, which may limit the sample size and representativeness. Future research should involve larger samples and prospective longitudinal studies to comprehensively understand the dynamic characteristics of social participation in young and middle-aged MG patients and its relationship with health outcomes.

## Data Availability

The original contributions presented in the study are included in the article/supplementary material, further inquiries can be directed to the corresponding author/s.
